# Standards for clinical trials for treating TB

**DOI:** 10.5588/ijtld.23.0341

**Published:** 2023-12-01

**Authors:** P. du Cros, J. Greig, J.-W. C. Alffenaar, G. B. Cross, C. Cousins, C. Berry, U. Khan, P. P. J. Phillips, G. E. Velásquez, J. Furin, M. Spigelman, J. T. Denholm, S. S. Thi, S. Tiberi, G. K. L. Huang, G. B. Marks, A. Turkova, L. Guglielmetti, K. L. Chew, H. T. Nguyen, C. W. M. Ong, G. Brigden, K. P. Singh, I. Motta, C. Lange, J. A. Seddon, B-T. Nyang’wa, A. K. J. Maug, M. T. Gler, K. E. Dooley, M. Quelapio, B. Tsogt, D. Menzies, V. Cox, C. M. Upton, A. Skrahina, L. McKenna, C. R. Horsburgh, K. Dheda, B. J. Marais

**Affiliations:** 1 Burnet Institute, Melbourne, VIC; 2 Monash Infectious Diseases, Monash Health, Melbourne, VIC, Australia; 3 Médecins Sans Frontières (MSF), Manson Unit, London, UK; 4 Sydney Infectious Diseases Institute (Sydney ID), and; 5 School of Pharmacy, Faculty of Medicine and Health, The University of Sydney, Sydney, NSW; 6 Westmead Hospital, Sydney, NSW; 7 Kirby Institute, University of New South Wales, Sydney, NSW, Australia; 8 Department of Pharmacology and Therapeutics, University of Liverpool, Liverpool; 9 Institute of Clinical Trials and Methodology, University College London, London, UK; 10 Interactive Research and Development Global, Singapore City, Singapore; 11 UCSF Center for Tuberculosis; 12 Division of Pulmonary and Critical Care Medicine, and; 13 Division of HIV, Infectious Diseases, and Global Medicine, University of California, San Francisco, San Francisco, CA; 14 Harvard Medical School, Department of Global Health and Social Medicine, Boston, MA; 15 Global Alliance for TB Drug Development, New York, NY, USA; 16 Victorian Tuberculosis Program, Melbourne Health, Melbourne, VIC; 17 Department of Infectious Diseases, Peter Doherty Institute for Infection and Immunity, University of Melbourne, Melbourne, VIC, Australia; 18 Eswatini National TB Control Program, Mbabane, Kingdom of Eswatini; 19 Blizard Institute, Barts and The London School of Medicine and Dentistry, Queen Mary University of London, London; 20 GlaxoSmithKline, London, UK; 21 Northern Health Infectious Diseases, Northern Health, Melbourne, VIC; 22 School of Clinical Medicine, University of New South Wales, Sydney, NSW, Australia; 23 Medical Research Council Clinical Trials Unit at University College London, London, UK; 24 Médecins Sans Frontières (MSF), Paris; 25 Sorbonne Université, Institut national de la santé et de la recherche médicale, Unité 1135, Centre d’Immunologie et des Maladies Infectieuses, Paris; 26 Assistance Publique Hôpitaux de Paris (APHP), Groupe Hospitalier Universitaire Sorbonne Université, Hôpital Pitié-Salpêtrière, Centre National de Référence des Mycobactéries et de la Résistance des Mycobactéries, Paris, France; 27 Department of Laboratory Medicine, National University Hospital, Singapore City, Singapore; 28 Research Department, Friends for International TB Relief, Ha Noi, Vietnam; 29 Infectious Diseases Translational Research Programme, Department of Medicine, National University of Singapore, Singapore City; 30 Division of Infectious Diseases, Department of Medicine, National University Hospital, Singapore City; 31 Institute of Healthcare Innovation & Technology, National University of Singapore, Singapore City, Singapore; 32 The Global Fund, Geneva, Switzerland; 33 Victorian Infectious Disease Unit, Royal Melbourne Hospital, Melbourne, VIC, Australia; 34 MSF, Geneva, Switzerland; 35 Division of Clinical Infectious Diseases, Research Center Borstel, Borstel; 36 German Center for Infection Research (DZIF), TTU-TB, Borstel; 37 Respiratory Medicine & International Health, University of Lübeck, Lübeck, Germany; 38 Baylor College of Medicine and Texas Children’s Hospital, Houston, TX, USA; 39 Department of Infectious Disease, Imperial College London, London, UK; 40 Desmond Tutu TB Centre, Department of Paediatrics and Child Health, Stellenbosch University, Tygerberg, South Africa; 41 Public Health Department, Operational Center Amsterdam (OCA), MSF, Amsterdam, The Netherlands; 42 Damien Foundation Bangladesh, Dhaka, Bangladesh; 43 De La Salle Medical and Health Sciences Institute, Dasmariñas, the Philippines; 44 Division of Infectious Diseases, Vanderbilt University Medical Center, Nashville, TN, USA; 45 Tropical Disease Foundation, Makati City, Manila, the Philippines; 46 KNCV Tuberculosis Foundation, The Hague, The Netherlands; 47 Mongolian Anti-TB Coalition, Ulaanbaatar, Mongolia; 48 Respiratory Epidemiology and Clinical Research Unit, Montreal Chest Institute & McGill International TB Centre, Montreal, QC, Canada; 49 Centre for Infectious Disease Epidemiology and Research, School of Public Health and Medicine, Faculty of Health Sciences, University of Cape Town, Cape Town; 50 TASK Applied Science, Cape Town, South Africa; 51 The Republican Scientific and Practical Center for Pulmonology and TB, Minsk, Belarus; 52 Treatment Action Group, New York, NY; 53 Departments of Global Health, Epidemiology, Biostatistics and Medicine, Schools of Public Health and Medicine, Boston University, Boston MA, USA; 54 Centre for Lung Infection and Immunity, Division of Pulmonology, Department of Medicine and UCT Lung Institute & South African MRC/UCT Centre for the Study of Antimicrobial Resistance, University of Cape Town, Cape Town, South Africa; 55 Faculty of Infectious and Tropical Diseases, Department of Immunology and Infection, London School of Hygiene & Tropical Medicine, London, UK; 56 The Children’s Hospital at Westmead, Sydney, NSW; 57 WHO Collaborating Centre in Tuberculosis, The University of Sydney, Sydney, NSW, Australia

**Keywords:** TB trials, tuberculosis, best practice, trial implementation

## Abstract

**BACKGROUND:**

The value, speed of completion and robustness of the evidence generated by TB treatment trials could be improved by implementing standards for best practice.

**METHODS:**

A global panel of experts participated in a Delphi process, using a 7-point Likert scale to score and revise draft standards until consensus was reached.

**RESULTS:**

Eleven standards were defined: Standard 1, high quality data on TB regimens are essential to inform clinical and programmatic management; Standard 2, the research questions addressed by TB trials should be relevant to affected communities, who should be included in all trial stages; Standard 3, trials should make every effort to be as inclusive as possible; Standard 4, the most efficient trial designs should be considered to improve the evidence base as quickly and cost effectively as possible, without compromising quality; Standard 5, trial governance should be in line with accepted good clinical practice; Standard 6, trials should investigate and report strategies that promote optimal engagement in care; Standard 7, where possible, TB trials should include pharmacokinetic and pharmacodynamic components; Standard 8, outcomes should include frequency of disease recurrence and post-treatment sequelae; Standard 9, TB trials should aim to harmonise key outcomes and data structures across studies; Standard 10, TB trials should include biobanking; Standard 11, treatment trials should invest in capacity strengthening of local trial and TB programme staff.

**CONCLUSION:**

These standards should improve the efficiency and effectiveness of evidence generation, as well as the translation of research into policy and practice.

## Abstract (fr)

**CONTEXTE:**

La valeur, la rapidité d’exécution et la solidité des données probantes générées par les essais de traitement de la TB pourraient être améliorées par la mise en œuvre de normes de bonnes pratiques.

**MÉTHODES:**

Un groupe mondial d’experts a participé à un processus Delphi, utilisant une échelle de Likert en 7 points pour noter et réviser les projets de normes jusqu'à ce qu’un consensus soit atteint.

**RÉSULTATS:**

Onze normes ont été définies: Norme 1, des données de haute qualité sur les régimes antituberculeux sont essentielles pour informer la gestion clinique et programmatique**;** Norme 2, les questions de recherche abordées par les essais sur la TB doivent être pertinentes pour les communautés affectées, qui doivent être incluses dans toutes les étapes de l’essai**;** Norme 3, les essais doivent s’efforcer d'être aussi inclusifs que possible**;** Norme 4, les conceptions d’essai les plus efficaces doivent être envisagées pour améliorer la base de données aussi rapidement et économiquement que possible, sans compromettre la qualité**;** Norme 5, la gouvernance des essais doit être conforme aux bonnes pratiques cliniques acceptées**;** Norme 6, les essais doivent étudier et rapporter les stratégies qui favorisent un engagement optimal dans les soins**;** Norme 7, lorsque cela est possible, les essais sur la TB doivent inclure des composantes pharmacocinétiques et pharmacodynamiques**;** Norme 8, les résultats doivent inclure la fréquence de la récurrence de la maladie et les séquelles post-traitement**;** Norme 9, les essais sur la TB doivent viser à harmoniser les résultats clés et les structures de données entre les études; Norme 10, les essais sur la TB doivent inclure des biobanques**;** Norme 11, les essais de traitement doivent investir dans le renforcement des capacités du personnel local chargé des essais et des programmes de lutte contre la TB.

**CONCLUSION:**

Ces normes devraient améliorer l’efficience et l’efficacité de la production de données probantes, ainsi que l’application de la recherche dans la politique et la pratique.

Momentum towards global TB elimination has been slow, with a reversal of progress during the COVID-19 pandemic.^1^ Both TB disease and post-TB disability disproportionately affect the poorest and most vulnerable.^2^ Improvements in the efficacy, tolerability, duration and overall cost of treatment are needed to curb the epidemic. In 2020, the global success rate for TB treatment was 86%, with no significant improvement in the preceding decade.^1^ For multidrug-resistant/rifampicin-resistant TB (MDR/RR-TB), treatment success was only 60%.^1^ Furthermore, there are few treatment options for people with TB, with delays in the introduction of improved therapies to special populations, such as children, pregnant people and people with HIV (PWHIV). High-quality evidence is needed to guide improvements to treatments for TB disease and prevention. In the past two decades, investment in TB treatment trials has increased, resulting in the approval of new drugs and regimens, enabling treatments to be shortened to 1 month for TB prevention, 4 months for drug-susceptible TB (DS-TB) and 6 months for drug-resistant TB. The clinical trials that ushered in these advances grappled with several challenges, of which the greatest continues to be inadequate funding, with the annual TB research funding gap estimated to be USD1 billion.^3^ TB treatment trials are particularly hampered by inadequate surrogate biomarkers for cure, requiring prolonged post-treatment monitoring for disease relapse. Learnings from fields like oncology has encouraged more innovative trial design, such as Bayesian adaptive randomised trials.

## AIM OF THE STANDARDS

Our aim is to present standards to complement the guidelines for good clinical practice (GCP) established by the International Council for Harmonisation of Technical Requirements for Pharmaceuticals for Human Use (ICH),^4^ to generate high-quality data, while also protecting the rights and safety of participants in therapeutic clinical trials. These standards also seek to ensure that key populations are not left behind and, in keeping with the IJTLD Clinical Standards for Lung Health,^5^–^12^ the standards support healthcare improvements by defining optimal levels of care. The standards are guiding principles for the development and implementation of TB therapeutic clinical trials, with the understanding that they might require context-specific adaptation.

## METHODS

We identified a global panel of experts representing clinical trialists, academics, civil society and implementers with TB programme knowledge and clinical trial experience. Special attention was given to ensure adequate regional representation and diversity. A total of 70 experts were invited and 40 responded within the specified time frame. A core group of eight people generated key domains for which potential standards could be developed and drafted initial statements that were then discussed and revised by the larger group. A 7-point Likert scale was used to assess agreement with initial draft statements (7 = high agreement; 1 = low agreement) and subsequent revisions. Standards were accepted if 90% of respondents assigned a score of 5 or higher; standards with less than 80% agreement were dropped and those with 80–90% agreement were reviewed. Additional standards and domains could also be suggested, and similar statements were consolidated as appropriate. A total of 13 domains and 39 statements were developed, and 11 standards were retained after multiple rounds of feedback and consensus building. Panel members were then invited to comment on the ‘final’ standards and accompanying text that constituted the draft manuscript. The final version was approved by all contributors with >90% consensus achieved for all standards.

## STANDARD 1

**High-quality data on TB regimens are essential to inform clinical practice and programmatic management.**

Recently updated WHO guidelines for the treatment of drug-resistant TB, including isoniazid-resistant and MDR/RR-TB, are based on low certainty of evidence.^13^ High-quality data refer to reliable and robust information reported from well-designed and meticulously conducted randomised controlled trials involving a sufficient and representative study population. Randomised controlled trials remain the gold standard for generating robust evidence to describe the benefits and harms of any health-related intervention. Long-term post-treatment follow-up assessments and transparent reporting of methods and results, as well as reproducibility, are vital. Also, careful consideration of the control or counterfactual comparator is required. Interpretation of trial results can be difficult if the control regimen is not standardised or is not the relevant counterfactual.

Although more investment in drug and regimen development is required, the TB drug development pipeline has improved considerably in recent years.^14^ Treatment of TB, however, requires effective regimens rather than individual drugs. When TB trials report results, it is important that all aspects in a regimen are considered, including which drugs were given, the duration, the dosage, the formulations and the delivery method used. Additionally, data on adherence, resistance, cost-effectiveness and acceptability are required. WHO target regimen profiles for TB treatment define 18 variables of regimens to be considered.^15^ It is imperative to generate high-quality evidence on regimens, including evaluation of all these aspects, for international and national guideline and policy processes to select the best regimens for programmatic use to achieve optimal treatment outcomes and epidemic control. Healthcare policies must be based on valid and reliable evidence to ensure that the highest standard of care is available to affected communities and that resources are allocated in an efficient, effective and equitable way. Alongside efficacy, effectiveness and acceptability data, persistent gaps in TB program funding means that cost-effectiveness data for new regimens are also vital. Although the directly observed therapy short-course (DOTS) strategy may have saved millions of lives, the use of empiric retreatment regimens based on limited evidence led to poor clinical outcomes, unnecessary toxicity, and likely, amplified drug resistance.^16^ To protect against the development of resistance to new treatment regimens, principles of good antibiotic stewardship should be incorporated, including considering the likelihood of resistance generation to regimens being trialled and, where required, the parallel development of appropriate drug susceptibility testing to support new treatment regimens.

## STANDARD 2

**The research questions addressed by TB treatment trials should be relevant to affected communities, who should be included in all trial stages from design to reporting.**

Community engagement is crucial for the success of clinical trials, especially in TB research, where obstacles such as stigma, limited healthcare access, social inequality and discrimination are common.^17^,^18^ There are limited initiatives to engage people with lived experience of TB and affected communities when adopting research priorities and designing therapeutic options. This results in missed opportunities to improve the social value, quality and impact of TB research. Inadequate engagement can have significant impact and can even contribute to the halting of trials, as has occurred with HIV trials.^19^ An MDR/RR-TB trial reported significant challenges with recruitment and retention,^20^ and potentially greater community engagement could have helped with improved strategies for some of the challenges. Potential misinformation and limited community knowledge of clinical trials underscore the pivotal role of involving community representatives in clinical trial planning and execution to combat mistrust.^21^,^22^ Historically, the absence of community perspectives from discussions of the potential benefits and harms of interventions being evaluated have enabled the routine use of suboptimal TB treatment regimens and exclusion of key groups from the TB research agenda and clinical trials.^23^

When communities are engaged early and thoroughly, they may ensure culturally appropriate study design, promote community sensitisation to current and future trials and facilitate acceptability, including enhanced trial recruitment and retention.^24^–^26^ Involvement of affected communities could also identify unique perspectives on research priorities. Currently, trials address research questions that investigators deem important (such as shortening treatment duration), while other aspects of treatment, such as pill burden or tolerability, may not be adequately considered. Formal measures should be built into trials on acceptability that could feed into evidence-to-decision frameworks used for guideline development. Additionally, community engagement in trials can support acceptance and uptake of successful novel treatment post-trial completion. Careful consideration should be given to post-trial treatment access for communities that participated in research where novel treatment regimens were shown to be effective. Finally, influential community gatekeepers could ensure there is appropriate collaboration with the community and would ensure that unethical practices, such as extracting information from participants without sharing trial findings, or insufficient consideration of participant burden, are avoided.^27^

Effective community engagement requires investment, equity-based and pragmatic approaches tailored to community priorities, needs and capacities (see Table 1). There may be differences of opinion (and even conflict) when engaging communities in research. Facilitation requires attention to maintain trustful, respectful relationships and awareness of power differentials and hierarchies.^28^ Key attributes of meaningful collaboration include early involvement of communities, acknowledgement of community expertise, cultural sensitivity, respect, and consideration of individual and community benefit.^24^ Thus, clinical trial investigators and sponsors should prioritise transparency and open communication, building trust through inclusive outreach and culturally appropriate engagement throughout the trial process.^29^

**Table 1:** Key concepts, recommendations and examples of strategies to effectively engage affected communities

Key concepts	Recommendations	Potential strategies
Community engagement in evidence generation and trial planning	Engage trusted community leaders, healthcare providers, patient advocates and stakeholders in clinical trial planning, design, governance, and implementation; Consult communities about research priorities, including acceptability of all aspects of treatment	Use existing strong community structures (e.g., community advisory boards, community health workers, women and youth groups, religious and traditional organisations) for trial planning; Establish functional community advisory groups or boards to advise and facilitate trial procedures; Conduct surveys prior to research to understand health priorities, access barriers, health-seeking behaviour to design effective engagement strategies
Capacity strengthening for access and participation	Develop context-specific, culturally sensitive materials to educate the community about trial importance, risks and benefits; Train and engage all health providers and research site staff to develop community engagement plans that consider cultural norms and communication preferences; Invest in clinical trial and community engagement infrastructure to increase access to all affected communities	Prioritise expanding access to clinical trials in rural, remote and underserved communities; Grade trials based on inclusive outreach and recruitment practices, rather than solely enrolment demographics, to better reflect recruitment equity; Establish trial procedures that allow use of digital platforms where possible, to enable telehealth consultations; Increase diversity through partnerships with communities, tailored support such as childcare, transportation, and meals, and the use of patient navigators and trusted voices in the community; Provide resources and support for participation, including assistance with travel and lodging expenses for participants and their families; Include funding to support local community engagement activities in research budgets; Empower and train affected communities to design and implement community-led research initiatives
Equitable data sharing, evidence dissemination and access to effective treatment	Recognise the expertise and contributions of community members and leaders and ensure active participation in data interpretation and results dissemination; Share data with communities to ensure that data generated by clinical trials benefit the communities they are collected from; Make successful regimens available to the communities and countries that participated in the trials	Empower and train affected communities to engage in disseminating trial findings and promoting access to successful regimens; Organise public events to provide opportunities for the community to engage with trial investigators and ask questions; Provide regular updates and feedback to the community about the trial progress; Revamp workflows, connect disparate systems, and recognise efficiencies that can improve the participant experience
Trust, transparency, and empowerment for equitable community engagement	Ensure open communication to build trust and deliver an excellent participant experience; Develop protocols that prioritise community ownership to ensure inclusive practices; Use respectful language and promote equitable participation; uphold the dignity of trial participants by avoiding stigmatising terms; Give people more choice and provide compensation for community expertise, as well as the cost associated with trial participation and retention	Tailor communication strategies specific to cultural norms and communication preferences of the target population; Establish interactive platforms or forums that facilitate routine and timely sharing of information, address community concerns, and foster greater communication and accountability between affected communities and investigators; Use technology and other innovative approaches as engagement tools to move beyond clinical trials as being transactional, focused only on collecting data

## STANDARD 3

**TB treatment trials should make every effort to be as inclusive as possible to increase the generalisability of the findings.**

There is both a clinical and ethical imperative for an inclusive approach in clinical trials. Ultimately, clinicians will need to treat all people who present for TB care and will need knowledge about safety and efficacy in as broad a range of people as possible to be able to do so. TB disease manifestations and burden, as well as pharmacokinetics and pharmacodynamics may vary among individuals with comorbidities (e.g., HIV, diabetes mellitus, renal disease, hepatitis or malnutrition), people who use drugs, pregnant/breastfeeding people, children and those with extrapulmonary TB. *Mycobacterium tuberculosis* strains can differ globally, with strain-specific differences in drug susceptibility or immune response,^30^ highlighting the importance of trials including sites on different continents. In people with TB and comorbidities (such as HIV and diabetes), drug interactions and overlapping toxicities may influence side effects, outcomes and choice of medications. From a clinical perspective, several considerations mean specific data are needed in a range of populations. Pharmacokinetics may differ depending on age, genetics, sex, pregnancy and weight extremes. The drug penetration and potency needed may differ in severe disseminated disease with high bacterial burden or in severe localised disease such as extensive lung cavities.^31^,^32^ Drugs need to cross the blood–brain barrier for central nervous system TB or to penetrate bones in TB osteomyelitis.^33^,^34^ Trials are needed to definitively answer the question posed by observational evidence that some forms of TB, which occur in immune privileged sites or sites with poor drug penetration (central nervous system and bone, for example), may require different durations of therapy, different drug combinations or adjunctive therapy. Yet, extrapulmonary TB manifestations (such as meningitis, osteomyelitis and disseminated TB) are often excluded from trials. While registries or smaller, specific trials are possible options, a solution is to recruit a representative cohort including these special groups. A clinical trial is the safest framework to support and protect participants.

Ethically there is a clear social justice requirement for broad-based access to clinical trials,^35^ as it encourages inclusion of those previously underserved by research for social and economic reasons. This includes persons incarcerated, migrants and those affected by conflict, all of whom frequently have a higher burden of TB. A disease of poverty such as TB requires inclusion of those from many backgrounds encompassing the true disease spectrum. It is important that vulnerable populations are not excluded from advances in science, but should have equity of access. For example, children, adolescents and pregnant people, who are supposed to be ‘protected’, have commonly been excluded from TB trials. This results in long delays before regimens that are approved for use in adults have sufficient evidence to be used or recommended. Adolescents with TB disease characteristics similar to adults are increasingly being included in adult TB trials, which can substantially reduce delays in use of improved regimens for this population.

The approach to inclusivity in trials does raise challenges, with broad inclusion likely to increase the complexity and potentially reduce the power to determine key outcomes due to increased heterogeneity. Furthermore, increasing inclusion will increase costs and can increase trial duration. Limited trial resources therefore favours the use of groups who are easier to recruit and retain, rather than a more representative range of participants. However, the resulting interventions may not be deliverable or beneficial to all groups within a population and further marginalise people with TB at highest risk of poor outcomes. Not only are such practices discriminatory,^36^ but they are also counterproductive and will leave a reservoir of persons with untreated TB. In balancing competing trial needs, it is important to consider the social determinants of disease and ensure those most needing improved TB treatment are not excluded from clinical trials.

## STANDARD 4

**The most efficient trial designs should be considered to improve the evidence base as quickly and cost-effectively as possible, without compromising quality.**

It has been recognised that observational, non-randomised studies are useful for evidence generation,^37^ and sophisticated causal inference methods have been developed^38^ that can yield less biased evidence from observational data.^39^ However, randomisation is the only method for balancing known and crucially unknown confounders, and for establishing an unbiased statistical framework for inference.^40^ This is universally recognised across disease areas and by regulatory bodies. People with TB deserve treatments based on the highest quality evidence.^41^

In 2022, funding for TB research first exceeded USD1 billion, but this remains far below what is needed.^3^ Given the urgency of reducing TB mortality and morbidity, clinical trials must utilise efficient designs that optimise available resources and generate high-quality evidence as rapidly as possible. Exploration of more innovative trial designs is a fruitful area of methodological research.^42^ Although TB research led randomised trial methodology with the development of innovations including concealed allocation in the 1940s,^43^ it is only now catching up with innovative clinical trial designs,^44^–^46^ including platform adaptive trials, seamless Phase II to III design and duration randomisation.^47^–^49^ Several categories of clinical trial design, highlighting the advantages and limitations of these for TB clinical trials, as well as potential contexts of use are shown in Table 2. The focus is on designs for late-stage clinical trials intended to provide confirmatory evidence for the efficacy and safety of a regimen and allow for registration and/or WHO policy generation. Other designs used for developing individual drugs (such as the ‘optimised background regimen design’^50^,^51^) are not covered here because TB disease is treated with combination regimens, not drugs in isolation. The present consensus statement encourages the use, where appropriate, of efficient designs including multi-arm randomised controlled design, multi-arm multi-stage design, Bayesian adaptive randomised design and duration evaluation design.

**Table 2:** Advantages and limitations of different clinical trial designs in assessing new TB treatment options

Design	Primary objective	Advantages	Limitations	Context of use
Uncontrolled cohort study	Quickly collect efficacy and safety data for a new regimen when there is no established standard of care	Smaller sample size due to absence of internal comparator; Operationally simple and pragmatic, as there is no randomisation, and all participants are treated with only a single combination regimen; Shorter time to completion	Requires use of a non-randomised external comparator, whereby unknown confounders cannot be balanced between arms; Participants often do better in clinical trials, further making comparisons problematic^103^	Necessarily a once-only design; Was a successful pathway for US Food and Drug Administration regulatory approval of a new regimen,^84^,^104^ but further observational data were required prior to WHO endorsement^105^
Two-arm randomised controlled design	Compare a new regimen against the standard of care	Straightforward design, represents the vast majority of clinical trials conducted in any disease^106^	Limited efficiency; only evaluates a single intervention; High-risk, given high likelihood of Phase 3 failure;^106^ Very slow progress in incorporating promising new treatment options	Investigator-initiated single centre-trials; Trials for extremely promising regimens for which it would be deleterious to wait for other interventions
Multi-arm randomised controlled design	Compare multiple new regimens against the standard of care	Higher chance of success with evaluation of more regimens; More public health focused in aiming to identify most promising regimen from a panel, rather than tied to a particular regimen; Less reliant on early-phase clinical trials based on suboptimal biomarkers for ruling out regimens	Large sample size; No opportunity to stop early or prioritise the most promising regimens based on early data; Requires collaboration of multiple owners of new drugs and regimens; Operationally complex at the pharmacy level and in managing side effects across a range of drugs	Well suited to TB regimen development when there are multiple regimens with potential for benefit where adaptations (see below) are not preferred
Multi-arm multi-stage design	Compare multiple new regimens against the standard of care, with early data being used to stop recruitment to poorly performing arms (based on pre-specified thresholds)	The same advantages as for multi-arm trials; Efficient use of resources by focusing on the most promising regimens; Participants enrolled in the study have an increasing likelihood of being allocated to more promising interventions; Sample sizes will be smaller than without adaptation; Permits oversight of the Data Monitoring Committee (DMC), taking into account all available data	Requires collaboration among multiple owners of new drugs; Operationally complex at the pharmacy level and in managing side effects across a range of drugs; Some complexity in trial design, but statistical methods and associated software are well established; Changes in patient population or disease epidemiology over time can introduce bias; Somewhat rapid data management systems necessary for scheduled interim analyses	Well suited to TB regimen development where the aim is to rapidly select among a panel of potentially promising candidates; Most suited where large spread in efficacy is not expected
Duration randomisation	Compare different treatment durations, using modelling to determine the optimal efficacious and safe duration of that regimen	Permits data-driven choices of treatment duration; De-risks the evaluation of shorter regimens	Adapting dose-finding methodology to duration-finding trials is novel and has not yet been prospectively validated; however, several trials are now planned or underway; Model-based analysis requires more analytic assumptions which may weaken evidence acceptance	Suited for novel regimens where there is limited data on which to base duration, and where there may be trade-offs between efficacy and safety
Strategy trial	Evaluate a patient management strategy that includes choice of regimen or modification, as well as other pragmatic aspects	Can be more programmatically relevant as it evaluates a specific regimen in the context of a strategy, rather than in isolation; Allows more autonomy/patient decisions to occur during trial; May permit more inclusive eligibility criteria and supportive patient management closer to usual standard of care, thus increasing generalisability; Often permits evaluation of considerably shorter durations than otherwise possible due to, for example, patient stratification, or consideration of alternative primary outcomes	Challenging to communicate results to stakeholders due to non-standard nature of interventions and design; Limitations will depend on specific design but might include reduced internal validity, leading to larger sample sizes; higher risk of adverse outcomes among some participants; delays due to lack of understanding by funders, regulators or ethics committees	Many contexts of use, depending on the type of strategy trial; often suitable for regimens shown to have promise in previous trials that need evidence to support implementation; Strategy trials will be crucial for generating high-quality evidence on impact of real-world implementation after a successful Phase 3 trial
Bayesian adaptive randomised design	Compare multiple new regimens against the standard of care, with early data being used to modify the randomisation– allocation ratio to progressively put more participants on better performing regimens	The same advantages as for multi-arm trials; can assess infinite number of treatment options; Efficient use of resources by focusing on the most promising regimens; Participants enrolled in the study have an increasing likelihood of being allocated to more promising interventions; Sample sizes will be smaller than without adaptation	May require collaboration of multiple owners of new drugs/regimens; Operationally complex at the pharmacy level and in managing side effects across a range of drugs; Some complexity in trial design, but statistical methods and associated software are well established; Most efficient when regimens include some that are promising and some that are not; Changes in patient population or disease epidemiology over time can introduce bias; Rapid data management systems essential for regular modifications to randomisation– allocation ratio	Well suited to TB regimen development where the aim is to rapidly select among a panel of potentially promising candidates

## STANDARD 5

**Trial governance should be in line with accepted good clinical practice.**

TB clinical trials require governance that adheres to internationally recognised guidelines and principles.^4^ Adhering to GCP standards from trial inception ensures scientific rigour with high quality of trial design and implementation, as well as data recording, verification and reporting. Particularly where a trial occurs in multiple countries, a robust GCP-based framework for oversight and conduct of the trial is crucial. Clinical trial governance in line with GCP helps ensure participant rights, safety and well-being.^52^ The COVID-19 pandemic facilitated increased use of digital tools and decentralised procedures, triggering revised recommendations for the European Union,^53^ but reinforcing the requirement for adherence to existing standards. Strict adherence to GCP standards are a requirement of regulatory authorities in many countries to accept data from clinical trials in support of marketing authorisation applications.^54^ Rigorous and ethically conducted trials help foster healthcare worker confidence and public trust in regimens demonstrating sufficiently successful outcomes.

## STANDARD 6

**Trials should investigate and report strategies that promote optimal engagement in care.**

Non-adherence to TB treatment increases the risk of treatment failure, relapse and acquisition of drug resistance.^55^,^56^ Participants who are lost to follow-up (LTFU) risk complete disengagement from healthcare, with high risk of unfavourable outcomes for individuals, and additional risks and implications for families and communities, including potential transmission of TB. Within a clinical trial context, high LTFU rates also risk the loss of substantive endpoint data, jeopardising trial integrity.^57^ Although statistical techniques can be applied to ameliorate the impact of missing data, the assumptions for imputing missing data may be incorrect, particularly if lost participants differ systematically from those retained.^58^ Phase III TB trials are particularly vulnerable due to the prolonged post-treatment follow-up periods.

Adherence is typically verified through treatment observation, but application of this observation can vary considerably. For example, DOT could refer to every dose being observed by a healthcare worker, trained lay provider, family member or via a plethora of digital technologies,^59^,^60^ and can be conducted in healthcare facilities or in the community. There has been increased research into treatment support, including trials of video directly observed treatment (V-DOT), electronic pill boxes and electronic reminders. However, as more options for treatment support become available, the best local solution may be highly context-specific.^61^ In addition, DOT is frequently practiced in trials differently to how it is implemented in programmatic settings, which can overestimate adherence and tolerability estimates that are not replicated in programmatic settings.

Trials do not always define how adherence was encouraged and assured, even when there may be concerns about drug intolerability. Strategies to reduce participant drop-out are similarly diverse and variably described. Common approaches include providing food security and financial incentives for every completed trial visit,^60^ and transportation to visits. Other strategies are to minimise in-person visits, replace in-person with digital visits or to have a permissive window for follow-up assessments. Unplanned strategies often develop during a trial’s evolution. For example, if a participant is LTFU, an unplanned strategy might be intensifying tracing through use of social media and/or visits to a participant’s home or village.^57^,^62^ Approaching community patient groups, especially diseases survivors, can prove beneficial for bringing back people who interrupt treatment and those not adhering to monitoring tests. Like other key aspects of a trial, the planned and unplanned adherence and participant retention strategies should be described in the trial protocol and trial outcomes reports.^57^,^59^,^63^

## STANDARD 7

**Where possible TB trials should include pharmacokinetic and pharmacodynamic components to generate information about treatment failure and toxicity.**

The relationship between treatment response (pharmacodynamics) and drug exposure (pharmacokinetics) is important to inform optimal treatment strategies. In early drug development, the parameter that correlates best with treatment response (for example, total drug exposure over time in relation to pathogen susceptibility) is identified, and exposure-response relationships for monotherapy are explored. In later-phase studies, pharmacokinetic data are important to understand factors that influence variability in exposures (e.g., sex, age or renal function). As some drugs display a high inter-patient variability in drug exposure, pharmacokinetic data can be important in exploring reasons why some treatments are less well-tolerated or less effective in certain populations,^64^,^65^ This is particularly the case in young children, pregnant people, or people with renal or hepatic dysfunction and older persons. It is especially worthwhile assessing drugs with a strong exposure-effect relationship.^66^ Including such analysis in Phase III studies, where there are remaining knowledge gaps, can have important implications for dosing strategies to improve patient outcomes.^67^,^68^ For example, finding the best tolerable dose of linezolid^69^,^70^ is an important subject of debate as cessation of linezolid (because of toxicity) may result in a less efficacious regimen. Including a pharmacokinetic assessment in the study protocol^67^,^70^,^71^ allows for a detailed secondary dose optimisation analysis. Exploring drug–drug interactions in trials is also important for component drugs of the TB regimen, or for drugs given for host-directed therapy or other indications (e.g., antiretroviral drugs).

Traditional pharmacokinetic assessments can be expensive and difficult to perform on many patients. Several new strategies can improve the feasibility and reduce the costs.^72^ Collecting fewer, but appropriately timed samples, can provide an adequate drug exposure assessment when combined with population pharmacokinetic modelling techniques.^73^ Instead of using single-drug assays for measuring drug concentrations, more advanced techniques can offer multi-analyte assays, thereby reducing laboratory costs.^74^ Costs for dry-ice shipments may be avoided by using dried blood spots, as demonstrated in HIV treatment trials,^75^ or by point-of-care saliva measurement using spectrophotometer assessment for drugs where this has been validated.^76^,^77^ The inclusion of participants of various ethnicities, as well as pharmacogenetic sub-studies may contribute to improved understanding of drug exposure, metabolism and toxicity.

## STANDARD 8

**Outcomes should include frequency of disease recurrence, as well as post-treatment sequelae.**

TB recurrence remains one of the most important endpoints for Phase III clinical trials for pulmonary TB. Inclusion of whole-genome sequencing can differentiate treatment relapse from re-infection and ideally should be routinely included in TB clinical trials.^78^ Recent trials have shown recurrence rates of 5–8% for the current 6-month DS-TB regimen,^79^–^82^ and most recurrence occurs within 6 months of completing DS-TB treatment. An analysis of 15 trials showed 78% and 91% of recurrence occurred within 6 and 12 months of stopping treatment, respectively.^83^ For MDR/RR-TB, 50–66% of recurrence has been reported within 6 months post treatment completion.^69^,^70^ Several recent TB trials have used a primary outcome equating to 6 months post completion of the longest treatment.^70^,^82^,^84^ Some regulatory authority guidelines concur with 6-month post-treatment for primary outcomes, whereas a 2-year follow-up post-treatment completion is recommended for secondary outcomes.^85^ The duration of follow-up can substantially contribute to TB trial duration and cost. Where trials evaluate regimens of different duration, the primary outcome should be at a fixed time-point from randomisation; shorter regimens will therefore have longer post-treatment follow-up. Nevertheless, follow-up of 6 months’ post-treatment completion, and ideally longer, is the recognised minimum.

Post-TB treatment sequelae can be either TB disease-related or persisting adverse drug events. Commonly these include post-TB lung disease, mental health disorders, visual impairment, hearing impairment, renal impairment and neurological impairment. The prevalence of respiratory impairment post-cure is estimated at 33% for DS-TB and 59% for MDR/RR-TB.^2^ Nearly half of the estimated 12.1 disability adjusted life-years due to incident TB are due to post-TB sequelae (5.8 DALYs).^86^ For extrapulmonary and culture-negative pulmonary TB, measures of microbiological cure are not currently possible. Outcome measures for trials involving these manifestations of TB will necessarily include post-treatment sequelae, with the specific measures dictated by the site (e.g., lung function, bone/joint deformity or neurological function post-meningitis).

In addition to physical impacts, TB has multidimensional and complex adverse effects on people’s psychological, economic and social well-being. Treatment generally improves quality-of-life (QOL) measures, although physical health frequently recovers before mental well-being. Impairment in QOL often remains post-treatment completion, with decreased work capacity, stigma and psychological issues.^87^,^88^ One third of TB-affected households suffer catastrophic costs,^89^ and frequently remain financially vulnerable post-treatment.^90^ How people affected by TB describe relevant post-TB sequelae requires further exploration. As TB treatment options increase, trade-offs between shorter duration, toxicity and efficacy require further consideration, including adequate assessment of post-treatment disability. For person-centred care, we must improve understanding of how treatment impacts QOL and how people with TB experience and report outcomes, further highlighting the need to involve affected communities in TB trials.

## STANDARD 9

**TB treatment trials should aim to harmonise key outcomes and data structures across studies to facilitate robust analyses of pooled data.**

Pooling data from clinical trials allows us to test hypotheses that are not possible with individual trials alone. Pooled studies of TB treatment are commonly performed through individual patient data meta-analysis (IPD-MA), allowing for more detailed evaluation of treatment outcomes than meta-analysis of summary effect estimates.^91^ IPD-MA has featured prominently in the development of WHO treatment guidelines for TB, beginning with an analysis of pooled observational cohorts to address research questions formulated by a WHO expert committee developing guidelines for treatment of MDR/RR-TB.^92^ IPD-MA has informed addition of new regimens to the guidelines, prioritisation of individual drugs in regimens, and permitted analyses of several key subgroups. IPD-MA has been extended from observational cohorts to clinical trials, facilitating development of a risk stratification algorithm that informed differentiated treatment approaches.^93^ Anonymised data sharing should be included within informed consent forms, with timely publication and sharing or placement in a public repository immediately after publication of primary trial results. This allows pooled analyses of the most up-to-date data for the common good.

In a recent systematic review of 31 pulmonary TB treatment trials, most trials used a composite binary outcome that combined treatment failure, relapse, death and treatment changes as ‘unfavourable’ in their primary efficacy outcome.^94^ An ‘unassessable’ category (e.g., LTFU, re-infection) was commonly used to exclude participants from particular analyses.^94^ Limitations of existing approaches are extensive and include a lack of standardisation that limits comparability, use of definitions incongruent with policy and guideline development groups, and conflation of safety and tolerability with efficacy in a single endpoint.^94^ The heterogeneity of key outcomes is not unexpected given the diverse field of investigators implementing trials with different objectives across continents, regulatory bodies and funders. Recent Phase IIC and Phase III randomised controlled trials and uncontrolled cohort studies all employed a composite primary endpoint, but with wide variability of key features, including definitions of treatment failure and relapse, the total duration of follow-up after randomisation (ranging from 6–30 months after randomisation), and definition of ‘unassessable’. An overview of key differences is shown in Supplementary Table S1. International efforts, such as the ICH +E9 (R1) addendum on estimands and sensitivity analyses in clinical trials, provide an approach to assist harmonisation of clinical trial design.^95^ The TB research, policy and patient community will greatly benefit if clinical trials harmonise key outcomes, including adverse events, and data structures, and make data available in a timely manner to facilitate robust analyses of pooled data.

## STANDARD 10

**TB treatment trials should include biobanking to add value from complementary studies.**

Biobanking is the process of acquiring and storing biological specimens with matched clinical data and patient meta-data in an organised system, with information on the activities related to collection, preparation, preservation, analysis and distribution of the biological specimens obtained.^96^,^97^ Biobanks provide well-characterised specimens for basic and translational research, facilitating early exploration, which could then be validated across geographically and environmentally different human populations.^98^ Investment in large, high-quality TB-related biobanks remains limited compared to cancer consortia around the world. In regions where the TB burden is high, legislative structures regulating the storage, use, dispersal and disposal of human biological samples, and harmonisation of these procedures, are often inadequate or non-existent.^99^ Furthermore, concerns relating to consent for unspecified future uses, as well as properly regulated access and data protection, are challenging. Clinical trials provide an excellent opportunity to establish the necessary regulatory framework to protect the interests of those who contribute to the biobank, whilst safeguarding the biological value inherent in these collections. Clinical trials that embed biobanking have the facility to collect samples in a standardised fashion, while gaining informed consent that prioritises the ethics, privacy and data security of those who choose to contribute.

One of the challenges of biobanks is the unspecified future use of specimens. Furthermore, trial funding is typically limited, with no capacity to support longer-term specimen storage, which differs from registry-linked, publicly funded centralised biobanks. However, trials that include complementary studies, which may be exploratory in nature, are often permitted to have biobanks linked into the trial. Specimens collected from clinical trial participants are highly valuable given randomised selection. It is important they are optimally utilised, including access to these samples by other investigators, to justify the generous donations made by participants and to recover the substantive costs associated with specimen collection and storage.

## Standard 11

**Treatment trials should invest in training and capacity strengthening of local trial and TB program staff.**

Clinical trials should include capacity strengthening efforts beyond skills acquired through other forms of research or routine programme strengthening.^100^ Staff participating in TB trials must be appropriately skilled for the varied tasks they will undertake, including GCP, participant recruitment and support, ethical and regulatory requirements, safety monitoring and reporting, confidential data management, specific trial procedures and trial management. Staff will be better equipped to conduct complex trials if supported through a participatory gap analysis followed by targeted investment to enhance their skills and knowledge prior to trial commencement. Throughout a trial there should be supervision, mentoring and opportunity for relevant staff to participate in reviews of trial progress, challenges, incidents and clinical event decisions.

Capacity strengthening should also aim to improve routine programme practices and enhance research capacity beyond immediate trial needs. There should be real efforts to identify, train and mentor investigators at sites with the long-term goal of enabling them to design and execute independent trials. Providing training opportunities to both trial-specific and TB programme staff may enhance cohesion and understanding for staff not directly involved in trial activities. Participating in and understanding the requirements of a rigorous trial deepens understanding of the evidence-base that informs treatment recommendations. Training with these broader benefits in mind could therefore improve patient care in the longer-term. Healthcare services may benefit through improved practices in standard procedures, infection control, laboratory services, documentation of care, communication about patient management and greater job satisfaction. Staff given opportunities to enhance their skills during a clinical trial may become future leaders or advocates who enable locally appropriate application of research findings, including driving health system and policy improvement. These investments should be relative to site capacity with due recognition of historical power and funding imbalances. Particularly at trial sites in low-resource settings, efforts should be prioritised to directly empower locally based researchers to have leading roles, offer quality career development opportunities and ensure effective information exchange so that site staff gain from being part of larger trial programmes.^101^ A community of skilled local researchers is key to increased capacity for pragmatic research focused on local priorities.^102^

## AREAS FOR FUTURE CONSIDERATION

Several areas of discussion within the expert group did not achieve consensus and require further consideration. For example, issues related to inclusion of disease manifestations with unique features (such as TB meningitis) may require specific evidence on optimal management, including drug penetration into sanctuary sites, appropriate markers of treatment success and consideration of targeted adjunctive treatment to minimise sequelae. Host-directed therapies could potentially contribute to improved clinical outcomes, shortened treatment duration and reduced post-treatment disability, and should be considered in future clinical trials. Consideration may be given to the role of surgery in addition to therapeutics in those with extensive cavitary disease. Evidence on relapse rates and post-TB sequelae with new regimens will also provide guidance for patient follow-up.

## CONCLUSION

Inadequate funding for TB trials hampers the pace of evidence generation and translation, and increases the global impact of TB on affected communities. The standards presented here aim to guide best practice in TB trial design and implementation to promote innovation and progress in support of the End TB Strategy. Incorporation of these standards may increase costs, but this should be offset by increased efficiency and utility. For example, the proposed standards should stimulate innovation, improve international collaboration and data sharing, as well as encourage greater involvement of affected communities. All these elements are essential to find solutions to reduce disease burden and achieve TB elimination.
