# Supplementary material for: Standards for clinical trials for treating TB
Source: Int J Tuberc Lung Dis. 2023 Dec 1;27(12):885–98. doi: 10.5588/ijtld.23.0341 (PMC10719894; doi:10.5588/ijtld.23.0341)
Supplement: Supplementary file 1 [file iutld_ijtld_23.0341_supplementarydata1.pdf]

http://dx.doi.org/10.5588/ijtld.23.0341

SUPPLEMENTARY DATA

Standards for clinical trials for treating TB

Supplementary Table S1. Range of key efficacy outcomes in completed and ongoing Phase 2C and Phase 3 tuberculosis randomised controlled trials and registrational cohort studies.

Trial name (registry number)	Years of active enrolment	Design	Primary endpoint †
**Drug-susceptible tuberculosis**			
DMID-01-009 (NCT00130247)[1]	2002-2007	Two-arm randomised controlled design	Bacteriological or clinical relapse at 30 months after the initiation of treatment.
OFLOTUB (NCT00216385)[2]	2005-2009	Two-arm randomised controlled design	Unfavourable outcome defined as the percentage of participants with treatment failure, recurrence (relapse or reinfection) or death at 24 months after the end of treatment, or withdrawal from the study during treatment. Treatment failure and recurrence were defined as two consecutive positive cultures at least 1 day apart. An independent endpoints committee unaware of study group assignment determined outcomes when the outcome could not be determined by the study team. Favourable outcome was defined as participants with negative cultures at the end of follow-up.
RIFAQUIN (ISRCTN44153044)[3]	2008-2011	Multi-arm randomised controlled design	Unfavourable outcome defined as the proportion of participants with a restart or change of treatment for any reason other than making up for missed doses or becoming pregnant, a positive culture from samples obtained at the most recent follow-up visit, death before the end of scheduled treatment for reasons other than violence or trauma, death after the end of treatment with evidence that confirmed or suggested possible treatment failure or relapse of their TB, and failure to complete treatment without a negative culture result at the end of the follow-up period. Relapse after completion of treatment was defined as two positive cultures within a period of 4 months

Trial name (registry number)	Years of active enrolment	Design	Primary endpoint †
			without an intervening negative culture and without genotypic evidence of reinfection. Favourable outcome was defined as having a negative culture at the scheduled end of follow-up and not previously classified as having an unfavourable outcome.
REMoxTB (NCT00864383)[4]	2008-2012	Multi-arm randomised controlled design	Unfavourable outcome defined as the proportion of participants with bacteriological or clinical treatment failure or relapse within 18 months after randomisation. Relapse strains were those shown to be identical on 24-locus MIRU analysis.
STAND/NC-006 (NCT02342886)[5]*	2015	Multi-arm randomised controlled design	Unfavourable outcome defined as the proportion of participants with bacteriological or clinical treatment failure or relapse 12 months after randomisation (from 50 to 54 weeks). Favourable outcome was defined as having a negative culture status (two consecutive negative cultures at least 1 week apart with no intervening positive result) at 12 months after randomisation, and not previously classified as having an unfavourable outcome.
S31/A5349 (NCT02410772)[6]	2016-2018	Multi-arm randomised controlled design	Favourable outcome defined as survival free of TB at 12 months after randomisation. Favourable status was assigned if a participant met all of the following criteria: was alive and free of TB at 12 months after randomisation; did not meet the criteria for unfavourable or not-assessable status; and had either an *M. tuberculosis*-negative result on the sputum culture at month 12,or at month 12 was unable to produce sputum or produced sputum that was contaminated but without evidence of *M. tuberculosis*. Unfavourable status was assigned if a participant had M. tuberculosis -positive cultures from two sputum specimens obtained at or after week 17 without an intervening negative culture, died or were withdrawn from the trial or lost to follow-up during the treatment period, had an *M. tuberculosis*-positive culture when last seen, died from TB during the post-treatment follow-up, or received additional treatment for TB.
TRUNCATE-TB (NCT03474198)[7]	2018-2020	Strategy trial	Unfavourable outcome defined as a composite of death before week 96 or ongoing TB treatment or active TB at week 96.
**Drug-resistant tuberculosis**			
STREAM Stage 1 (NCT02409290)[8]	2012-2015	Two-arm randomised controlled design	Favourable outcome defined as cultures negative for *M. tuberculosis* at 132 weeks after randomisation and at a previous occasion during the trial period, with no intervening positive culture or previous unfavourable outcome. Unfavourable outcome defined by the initiation of two or more drug therapies that were not included in the assigned regimen, treatment extension beyond the permitted duration, death from any cause, a positive culture

Trial name (registry number)	Years of active enrolment	Design	Primary endpoint †
			from one of the two most recent specimens, or no visit at 76 weeks or later. Participants who had reinfections with a different strain and those whose last two cultures were negative (including one at 76 weeks) but were lost to follow-up thereafter were considered to be unable to be assessed and were excluded from the primary analysis.
NExT (NCT02454205)[9]	2015-2019	Two-arm randomised controlled design	Favourable outcome defined according to the WHO 2013 definition (sum of cured and treatment completed) without any events defining an unfavourable outcome (including treatment failure, relapse, reinfection, loss to follow-up, or death) 24 months after initiation of treatment.
Nix-TB (NCT02333799)[10]	2015-2017	Uncontrolled cohort study	Unfavourable outcome defined as treatment failure (bacteriological or clinical) or disease relapse. Clinical treatment failure was defined as a change from the protocol-specified TB treatment as a result of a lack of clinical efficacy, retreatment for TB, or TB-related death through follow-up until 6 months after the end of treatment. Favourable outcome defined as resolution of clinical TB disease, negative culture status at 6 months after the end of therapy, and not previously classified as having an unfavourable outcome.
STAND/NC-006 (NCT02342886)[5*]	2015	Uncontrolled cohort study	Unfavourable outcome defined as the proportion of participants with bacteriological or clinical treatment failure or relapse 12 months after randomisation (from 50 to 54 weeks). Favourable outcome defined as having a negative culture status (two consecutive negative cultures at least 1 week apart with no intervening positive result) at 12 months after randomisation, and not previously classified as having an unfavourable outcome.
MDR-END (NCT02619994)[11]	2016-2019	Two-arm randomised controlled design	Favourable outcome defined according to the WHO 2014 definition (sum of cured and treatment completed) without any events defining an unfavourable outcome (including treatment failure, relapse, death, loss to follow-up, transfer out, withdrawal) 24 months after initiation of treatment. Unassessable conditions included death, loss to follow-up, transfer out, withdrawal, reinfection after treatment completion or culture conversion, or withdrawal due to pregnancy.
STREAM Stage 2 (NCT02409290)[12]	2016-2020	Multi-arm randomised controlled design	Favourable outcome defined as a negative culture for *M. tuberculosis* at week 76 and on the preceding visit, with no intervening positive culture or previous unfavourable outcome. Unfavourable outcome defined as treatment initiation with bedaquiline, kanamycin, linezolid, or two or more other drugs if they were not part of the assigned regimen; treatment extension beyond the permitted duration; death from any cause; a positive culture from one of the two most recent specimens; or no week 76 visit.

Trial name (registry number)	Years of active enrolment	Design	Primary endpoint †
ZeNix (NCT03086486)[13]	2017-2019	Multi-arm randomised controlled design	Unfavourable outcome defined as treatment failure (bacteriological or clinical) or disease relapse at 26 weeks after completion of treatment. Bacteriological treatment failure was defined as negative culture status not attained or maintained during treatment. Clinical treatment failure was defined as a change from the protocol-specified TB treatment as a result of a lack of clinical efficacy, retreatment for TB, or TB-related death.
endTB (NCT02754765)[14]	2017-2021	Bayesian adaptive randomised design	Favourable outcome defined as not previously classified as unfavourable by week 73, and one of the following is true: [1] the last two culture results are negative. These two cultures must be taken from sputum samples collected on separate visits, the latest between weeks 65 and 73; or [2] the last culture result (from a sputum sample collected between weeks 65 and 73) is negative, and either there is no other post-baseline culture result or the penultimate culture result is positive due to laboratory cross contamination, and bacteriological, radiological and clinical evolution is favourable; or [3] there is no culture result from a sputum sample collected between weeks 65 and 73 or the result of that culture is positive due to laboratory cross contamination, and the most recent culture result is negative, and bacteriological, radiological, and clinical evolution is favourable.
TB-PRACTECAL (NCT02589782)[15]	2017-2021	Phase 2-3, multi arm multi-stage randomised controlled trial	Unfavourable status was defined as a composite of death, treatment failure, treatment discontinuation, loss to follow-up, or recurrence of TB at 72 weeks after randomization
BEAT-India (CTRI/2019/01/017 310)[16]	2019-2021	Uncontrolled cohort study	Favourable outcome defined as 2 consecutive sputum cultures taken at least 4 weeks apart were negative, with clinical and radiological improvement at the end of treatment.
endTB-Q (NCT03896685)[14]	2020-2023	Strategy trial	Favourable outcome defined as the proportion of participants whose outcome is not classified as unfavourable at week 73, and for whom one of the following is true: the last two culture results are negative (these two cultures must be taken from sputum samples collected on separate visits, the latest between week 65 and week 73); the last culture result (from a sputum sample collected between week 65 and week 73) is negative, and either there is no other post-baseline culture result or the penultimate culture result is positive due to laboratory cross contamination, and bacteriological, radiological and clinical evolution is favourable; or there is no culture result from a sputum sample collected between week 65 and week 73 or the result of that culture is positive due to laboratory

Trial name (registry number)	Years of active enrolment	Design	Primary endpoint †
			cross contamination; and the most recent culture result is negative; and bacteriological, radiological and clinical evolution is favourable.
DRAMATIC (NCT03828201)	2022-present	Duration evaluation	Favourable outcome defined as sustained cure at 76 weeks after randomisation without treatment failure or relapse.

Abbreviations: MIRU, mycobacterial interspersed repetitive unit.

* These trials are listed under both the drug-susceptible and drug-resistant sections due to inclusion of these two populations. In each case, study design differed for the drug-susceptible and drug-resistant populations.

† The primary endpoints are listed here. Many trials had multiple secondary endpoints, with much longer follow up periods than the primary endpoints.

References

1. Johnson JL, Hadad DJ, Dietze R, Noia Maciel EL, Sewali B, Gitta P, et al. Shortening Treatment in Adults with Noncavitary Tuberculosis and 2-Month Culture Conversion. Am J Respir Crit Care Med. 2009 Sep 15;180(6):558–63.
2. Merle CS, Fielding K, Sow OB, Gninafon M, Lo MB, Mthiyane T, et al. A Four-Month Gatifloxacin-Containing Regimen for Treating Tuberculosis. N Engl J Med. 2014 Oct 23;371(17):1588–98.
3. Jindani A, Harrison TS, Nunn AJ, Phillips PPJ, Churchyard GJ, Charalambous S, et al. High-Dose Rifapentine with Moxifloxacin for Pulmonary Tuberculosis. N Engl J Med. 2014 Oct 23;371(17):1599–608.
4. Gillespie SH, Crook AM, McHugh TD, Mendel CM, Meredith SK, Murray SR, et al. Four-Month Moxifloxacin-Based Regimens for Drug-Sensitive Tuberculosis. N Engl J Med. 2014 Oct 23;371(17):1577–87.
5. Tweed CD, Wills GH, Crook AM, Amukoye E, Balanag V, Ban AYL, et al. A partially randomised trial of pretomanid, moxifloxacin and pyrazinamide for pulmonary TB. Int J Tuberc Lung Dis. 2021 Apr;25(4):305–14.
6. Dorman SE, Nahid P, Kurbatova EV, Phillips PPJ, Bryant K, Dooley KE, et al. Four-Month Rifapentine Regimens with or without Moxifloxacin for Tuberculosis. N Engl J Med. 2021 May 6;384(18):1705–18.
7. Paton NI, Cousins C, Suresh C, Burhan E, Chew KL, Dalay VB, et al. Treatment Strategy for Rifampin-Susceptible Tuberculosis. N Engl J Med. 2023 Mar 9;388(10):873–87.
8. Nunn AJ, Phillips PPJ, Meredith SK, Chiang CY, Conradie F, Dalai D, et al. A Trial of a Shorter Regimen for Rifampin-Resistant Tuberculosis. N Engl J Med. 2019 Mar 28;380(13):1201–13.
9. Esmail A, Oelofse S, Lombard C, Perumal R, Mbuthini L, Goolam Mahomed A, et al. An All-Oral 6-Month Regimen for Multidrug-Resistant Tuberculosis: A Multicenter, Randomized Controlled Clinical Trial (the NExT Study). Am J Respir Crit Care Med. 2022 May 15;205(10):1214–27.

10. Conradie F, Diacon AH, Ngubane N, Howell P, Everitt D, Crook AM, et al. Treatment of Highly Drug-Resistant Pulmonary Tuberculosis. N Engl J Med. 2020 Mar 5;382(10):893–902.
11. Mok J, Lee M, Kim DK, Kim JS, Jhun BW, Jo KW, et al. 9 months of delamanid, linezolid, levofloxacin, and pyrazinamide versus conventional therapy for treatment of fluoroquinolone-sensitive multidrug-resistant tuberculosis (MDR-END): a multicentre, randomised, open-label phase 2/3 non-inferiority trial in South Korea. The Lancet. 2022 Oct 29;400(10362):1522–30.
12. Goodall RL, Meredith SK, Nunn AJ, Bayissa A, Bhatnagar AK, Bronson G, et al. Evaluation of two short standardised regimens for the treatment of rifampicin-resistant tuberculosis (STREAM stage 2): an open-label, multicentre, randomised, non-inferiority trial. The Lancet. 2022 Nov 26;400(10366):1858–68.
13. Conradie F, Bagdasaryan TR, Borisov S, Howell P, Mikiashvili L, Ngubane N, et al. Bedaquiline–Pretomanid–Linezolid Regimens for Drug-Resistant Tuberculosis. N Engl J Med. 2022 Sep 1;387(9):810–23.
14. Guglielmetti L, Ardizzoni E, Atger M, Baudin E, Berikova E, Bonnet M, et al. Evaluating newly approved drugs for multidrug-resistant tuberculosis (endTB): study protocol for an adaptive, multi-country randomized controlled trial. Trials. 2021 Sep 25;22(1):651.
15. Nyang'wa BT, Berry C, Kazounis E, Motta I, Parpieva N, Tigay Z, et al. A 24-Week, All-Oral Regimen for Rifampin-Resistant Tuberculosis. N Engl J Med. 2022 Dec 22;387(25):2331–43.
16. Padmapriyadarsini C, Vohra V, Bhatnagar A, Solanki R, Sridhar R, Anande L, et al. Bedaquiline, Delamanid, Linezolid and Clofazimine for Treatment of Pre-extensively Drug-Resistant Tuberculosis. Clin Infect Dis Off Publ Infect Dis Soc Am. 2022 Jun 29;76(3):e938-946.
